# Documenting a cultural landscape using point-cloud 3d models obtained with geomatic integration techniques. The case of the El Encín atomic garden, Madrid (Spain)

**DOI:** 10.1371/journal.pone.0235169

**Published:** 2020-06-24

**Authors:** Tomás Ramón Herrero-Tejedor, Francisco Arqués Soler, Serafín López-Cuervo Medina, Manuel Rodrigo de la O Cabrera, Juan Luis Martín Romero

**Affiliations:** 1 Department of Agroforestry Engineering, Universidad Politécnica de Madrid, Madrid, Spain; 2 Department of Architectural Design, Universidad Politécnica de Madrid, Madrid, Spain; 3 Department of Surveying and Cartography Engineering, Universidad Politécnica de Madrid, Madrid, Spain; 4 Department of Architectural Composition, Universidad Politécnica de Madrid, Madrid, Spain; Indiana State University, UNITED STATES

## Abstract

A country’s cultural landscapes are an important part of its heritage. The growing need to identify, catalogue and preserve these resources has led to a rapid change in the management and inventorying of heritage in general and of cultural landscapes in particular. The main aim of this work is to develop and apply an updated and integrated methodology for capturing and processing geo-information for the digital documentation of cultural heritage. The proposed case study is the atomic garden in the Finca El Encín (Madrid), a singular space with unique biogeographical features created over 60 years ago. The results of the case study validate the method, consisting of an unmanned aerial platform equipped with sensors to obtain point clouds and aerial images in conjunction with point clouds and images captured with a terrestrial laser scanner.

## 1. Introduction

### 1.1. Definition and context

Since the ratification of the European Landscape Convention [1], a growing number of cultural landscapes have been included in conservation policies in Spain. The aim of the European Landscape Convention is to promote the protection, management and planning of European landscapes, and the signatory countries undertake to “recognise landscapes in law as an essential component of people’s surroundings, an expression of the diversity of their shared cultural and natural heritage, and a foundation of their identity”. In 2012, the Spanish state approved the National Cultural Landscape Plan (PNPC) which defines this resource as “the result of the interaction between people and the natural environment over time, whose expression is a territory perceived and valued for its cultural qualities, the product of a process, and a cornerstone of a community’s identity” [2]. Although the plan incorporates the fundamental ideas of the European Landscape Convention, it is essentially inspired by the UNESCO World Heritage Convention [3–4], and therefore prioritises conservation actions in landscapes of outstanding cultural interest.

Cultural landscapes are dynamic and complex environments that generally cover large extensions of territory. The regulation recommends the adequate delimitation of their management possibilities so they can be handled as a cultural resource [5]. They are identified in terms of the size of their core zone, and may range from a very large historic garden to territorial systems with an area of between 100 and 2,500 ha (with certain exceptions); this represents a substantial quantitative increase in the actual physical space in need of conservation and requires the appropriate methods and instruments [6].

This quantitative increase in cultural resources has also led to a qualitative shift. Conservation theory and practice has traditionally been based on the stability of the material. However, attempts to include cultural landscapes on the World Heritage List highlighted the difficulty of applying the principle of authenticity based on their material stability; on the contrary, landscape was recognised as a complex and diverse form of heritage within which “it is important that due weight be paid to the full range of values represented in the landscape, both cultural and natural” [7]. The problems involved in assessing cultural resources are therefore not merely due to the veracity of tangible remains, but also to their perception and interpretation.

### 1.2. Documenting the existing conditions in a cultural landscape

The first step in the conservation of any cultural resource or landscape is the meticulous documentation of its existing features. This can then be used as the baseline for the subsequent historical characterisation of aspects such as spatial organisation, visual relations, circulation systems, land use, topography, vegetation, water, buildings and structures, and objects. It is also a key factor in any assessment, treatment or management actions [8–10].

One of the first aspects to consider when documenting the characteristics of the cultural landscape is its territorial dimension. While it is important to choose a scale that is sufficiently precise to describe the arrangement of vegetation elements, architecture, topography and water flows [11], too much information may make it difficult to gain a clear reading of the basic features of the spatial, functional and visual layout of the site. One common solution is to individually document unique areas within a landscape that require greater accuracy.

A second aspect is the variety of information. The PNPC (2012) concurs with the international regulation by specifying the detailed documentation of at least the physical environment (soil study, physiognomy, hydrology, flora and fauna), manmade elements (buildings and infrastructures), spatial organisation and its perceptive potential [12–13]. To collect this wide range of information the work is usually divided among professionals and managers from different disciplines, and the resulting partial information is then amalgamated in an inventory [14–15].

The inclusion of living material is a third aspect. Vegetation requires working without the material stability typical of built or movable heritage [16–17]. Although there is certainly a precedent in the conservation of historic gardens, landscapes are spatially more extensive, dynamic and changing systems [18] whose conservation is not always associated to a set pattern of plantations, and may apply intervention criteria that are adapted to complex socio-ecological relations on a territorial scale [19–20]. It is therefore important to identify and catalogue each vegetation element and include a statistical analysis to identify species, situation and distribution, state of vitality, age distribution and spatial characteristics (height, volume, alignment etc.) [21].

### 1.3. Motivation

A wide variety of techniques can be used to document the characteristics of a cultural landscape, including aerial photographs, maps, sections, narrative descriptions, photographs and videos [22–26]. In recent years there has been a growing tendency to use panoramic photographs, 360° photographs and videos [27], which have proved highly effective for an immersive analysis of visual quality. A 3D point cloud can represent the cultural features in its surrounding landscape and capture reference information for subsequent processing or management phases [28–31]. A frequent practice since the origins of landscape planning has been to compile inventories of documentation containing data in different formats, depending on the discipline contributing them and on the nature of the information. This involves painstaking fieldwork to obtain this documentation followed by the laborious task of compiling the information, whereas non-intrusive geomatic techniques currently have the potential to perform most of these tasks in a more economical, detailed and integrated way [32–34].

The proposed acquisition method in this research, with UAV and TLS combined systems, detects the physical identity of all biotic or inert elements, in addition to the basic ecological characteristics of the vegetation. The resulting spatial model also admits a wide range of scales, delivers all the data and simplifies the handling of more detailed information, while providing a complete and integrated immersive representation of the landscape.

Two specific criteria stood out when preparing to document the Atomic Garden: first, the wide variety of tree species, including Canadian poplar, acacia, cypress, maple, black poplar, fir, cedar, paradise tree, catalpa and privet; and second, the fact that this is a compact space with clearly defined boundaries, making it easy to measure the parameters of its spatial organisation.

Based on these factors, the proposed methodology was developed to cover the following objectives:

To obtain a suitable procedure for documenting the characteristics of a cultural landscape through a point-cloud model [35–36]. This procedure was verified on a complex fragment of a cultural landscape with information acquired by an unmanned aerial vehicle (UAV) and terrestrial laser scanning (TLS) [37–38]. The aim is to acquire precise, rapid and easily extrapolable documentation using inexpensive methods [39], This research describes a methodology of how the biotic as well as abiotic elements can be mapped using UAV and TLS in cultural heritage areas [40].

## 2. Materials and methods

### 2.1. Study area

The case study selected is a former gamma ray radiation field of 15.2 ha that was used between 1961 and 1973 to test nuclear energy applications for agriculture, and popularly known as the “atomic garden” [41]. The cultural value of this rare element of agrifood heritage derives from its historical singularity and the originality of its design.

**Fig 1:**
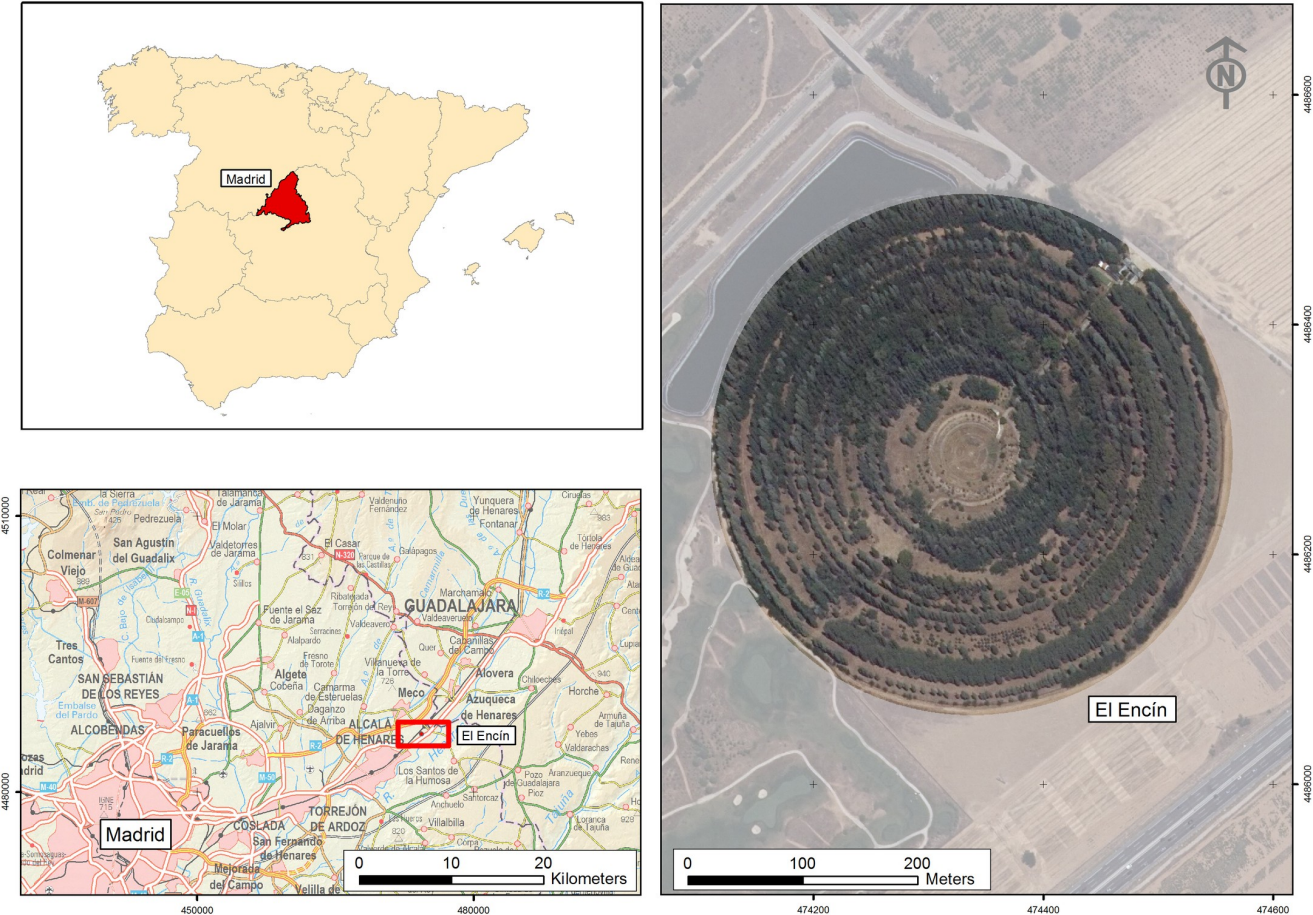
Location of the cultural landscape of the El Encín atomic garden (Madrid). UTM-30N-ETRS89. BCN200 2018 CC-BY 4.0 ign.es.

The garden is actually only a fragment of the cultural landscape of the El Encín agrifood research facility, located 40 km east of Madrid. It was chosen as the study site as it is the part of the premises that is most difficult to document owing to its layout, density and its varied tree cover. Good results in this area would support the potential application of the method to the rest of the landscape. (Fig 1).

The garden is arranged in two zones of concentric circles. The first, the experimentation field itself, is a circle measuring 50 m in diameter with the emission source in the centre. The second zone is a copse designed to serve as a protective barrier and formed by a succession of 24 rings of trees with a final diameter of 440 m. The two zones are separated from each other by a protective embankment with a triangular section measuring 10 m wide and 4 m high.

The garden has a single access point to the northeast of the site. A straight, 3 m-wide path leads from the entrance through the tree stands and ends at the central experimentation zone. This path corresponds to one of the spokes of the garden and is only intersected by a zigzag safety crossing on the ring dividing the central zone and the tree stand. There is a small office and a laboratory next to the entrance to the garden [42].

### 2.2. Material

Geospatial data were captured with UAV, TLS and GNSS (Global Navigation Satellite System). The equipment and materials used in these captures are described below, along with the characteristics of use of each one.

#### 2.2.1. Photogrammetric equipment

Photographs were captured by UAV with two sensors: One with the RGB Phantom 4 camera and another with Micasense RedEdge multispectral camera. (Table 1).

**Table 1:** Specifications and data collected with UAV systems.

	RGB Flight	Multispectral flight
RPAS	DJI Phantom4	DJI Phantom4
Camera	DJI FC330	Micasense RedEdge
Resolution (px)	12Mpx (4000x3000 px)	1.2Mpx (1280x960px)
Flight height (m)	80	82m
GSD (cm)	3.3	5.63
Passes and images	Passes 7, total 411 images	Passes 8, total 387 images
Overlap (forward & pass)	F 70% & P 60%	F 70% & P 60%
Bands	RGB (3 bands)	Multispectral (B, G, R, RE, NIR)

There were programmed two flights, first one for RGB at height of 80m with a GSD of 3.3cm. The second for the multispectral information at 82m with a GSD of 5.6cm. RGB camera allowed to study the dimensions of vegetation structure and the multispectral one allowed to calculate vegetation indexes (VI) such as NDVI and NDRE capable of estimating both vegetative vigor and the health of the vegetative coverage. [43–45].

#### 2.2.2. TLS equipment

TLS technology enables very dense point clouds to be obtained from the ground [46].

Fifty scan positions were established with the TLS equipment, covering the entrance, main access path to the centre of the garden, the centre itself and two areas of trees differentiated by the density of stems or trees per m^2^. Two methods of capture were used: a polygon with stations every 20 m for the access path; and a grid capture in the centre of the garden and the wooded areas in order to eliminate areas of shadow in the scan and capture the trees from all the different possible visuals. The parameters for the capture are shown in Table 2.

**Table 2:** TLS specifications and capture parameter setup.

	TLS
Equipment	Faro Focus S330
Range (m)	0.6–330
Field of view	300º / 360º
Camera resolution	Up to 70Mpx (360º)
Capture resolution (mm)	7mm at 10m
Accuracy (mm)	0.3 at 25m (90% Refl.)
	0.5 at 25m (10% Refl.)
Laser classification	Laser Class 1

#### 2.2.3. GNSS equipment

**Fig 2:**
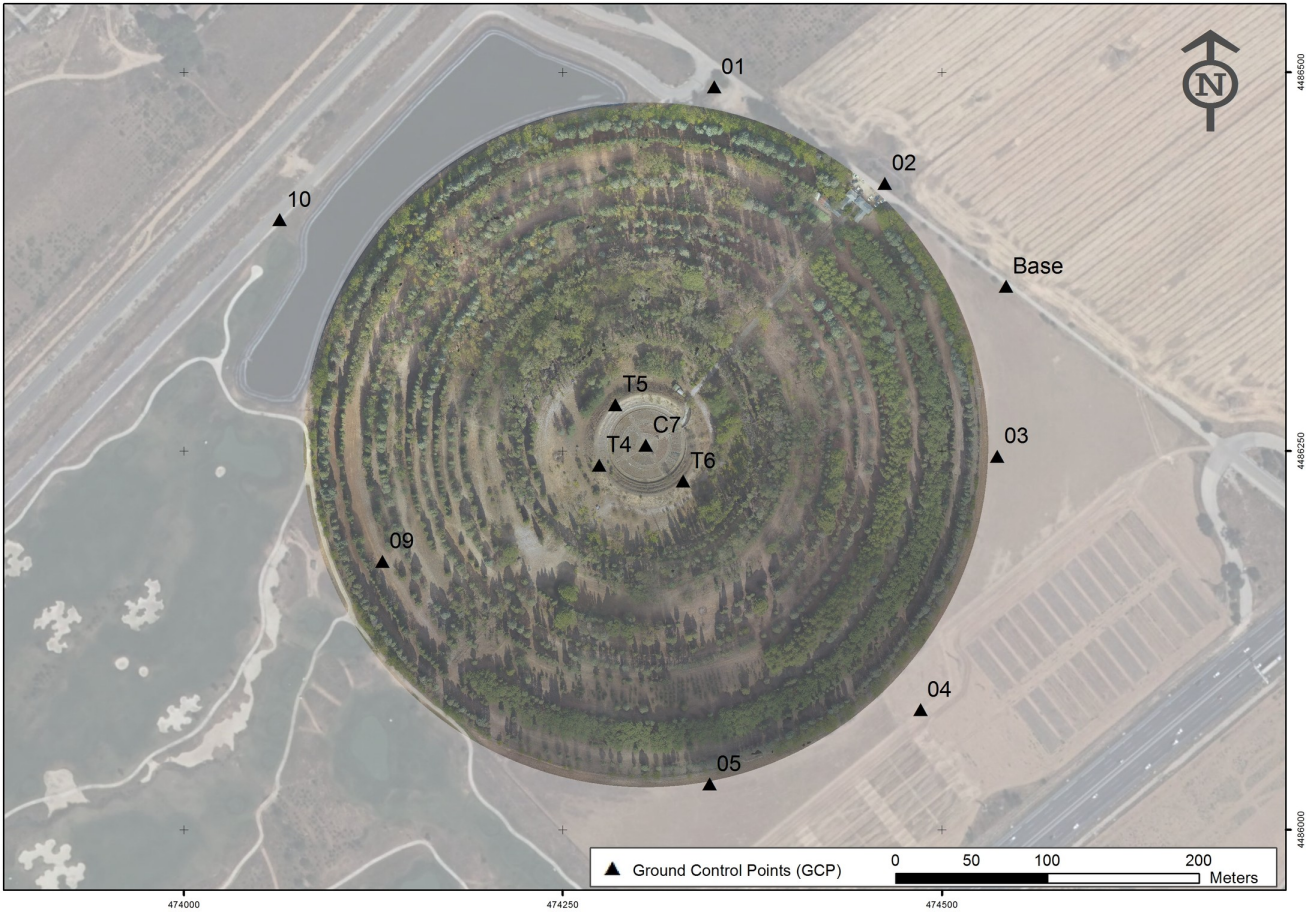
Distribution of GCPs for GPS–RTK in the field.

The garden was completely geo-referenced using GNSS equipment, combining the point cloud scanned with TLS and creating the framework of reference for the UAV photographs and their products (Fig 2) based on 14 40 x 40-cm targets (chessboard design). These GCPs (Ground Control Points) were measured with GPS RTK (Global Positioning System–Real-Time Kinematic) and served as pre-signalled GCPs for the UAV [47]. The TLS data were adjusted with GCPs 02 and C7, and the results are shown in Table 3.

**Table 3:** Error calculation in the GCP.

CODE	STATION	COORDINATES			PROJ.	STANDARD DEVIATION		
TYPE	NAME	X (m)	Y (m)	ELEV. (m)		X (m)	Y (m)	ELEV. (m)
Base	Encin	474542.670	4486358.930	604.564	UTM 30	0.006	0.004	0.012
DIANE	1	474349.945	4486490.564	604.934	UTM 30	0.017	0.013	0.031
DIANE	1B	474356.708	4486485.164	604.938	UTM 30	0.009	0.006	0.015
DIANE	2	474462.611	4486426.868	605.081	UTM 30	0.008	0.006	0.015
DIANE	3	474536.630	4486246.772	603.722	UTM 30	0.017	0.013	0.029
DIANE	4	474486.019	4486079.487	603.198	UTM 30	0.007	0.005	0.014
DIANE	5	474346.916	4486030.407	602.799	UTM 30	0.019	0.012	0.035
DIANE	9	474131.033	4486177.350	603.200	UTM 30	0.027	0.013	0.038
DIANE	10	474063.070	4486402.887	603.549	UTM 30	0.012	0.008	0.023
DIANE	14	474156.338	4486133.839	603.028	UTM 30	0.009	0.006	0.017
DIANE	15	474111.613	4486237.748	603.501	UTM 30	0.008	0.008	0.016
DIANE	C7	474304.802	4486254.018	603.048	UTM 30	0.016	0.010	0.027
DIANE	T4	474274.099	4486240.879	606.832	UTM 30	0.007	0.005	0.014
DIANE	T5	474284.704	4486280.743	606.866	UTM 30	0.010	0.005	0.016
DIANE	T6	474329.412	4486230.382	606.802	UTM 30	0.008	0.005	0.015
REF.	YEBE	492492.886	4486022.150	920.823	UTM 30	0.000	0.000	0.000
REF.	IGNE	439830.797	4477484.240	715.807	UTM 30	0.000	0.000	0.000

### 2.3. Methodology: Workflow. Geomatic techniques

Methods based on high-definition three-dimensional models have been applied for the conservation, management and dissemination of moveable and permanent cultural resources with considerable success [48]. Similar digital models are also increasingly used for research or design in environmental sciences and landscape architecture [49–52]. Three-dimensional models are considered essential for geovisualisation and representation of trees canopies, and enable the comparison and integration of TLS and UAV techniques

In the specific case of the atomic garden (former gamma radiation field), priority was given to capturing information on the spatial conditions of the elements and vegetation. This specific aspect–which is typical of cultural landscapes–serves as the basis for this methodology, in addition to other complementary issues such as determining the species present and their state of vitality with field radiometry.

The methodology consists of six differentiated but complementary parts.

The first phase (Phase 1) consists of reviewing and collecting the available data on the study site. This open-source information is accessible to the general public and researchers in particular, and mainly comprises documents, old maps, plans and maps from the National Aerial Orthography Plan (PNOA), and point clouds from light imaging detection and ranging (LIDAR) flights. These data serve as a graphic document that offers an initial approach to the configuration of this cultural landscape and its subsequent evolution.

**Fig 3:**
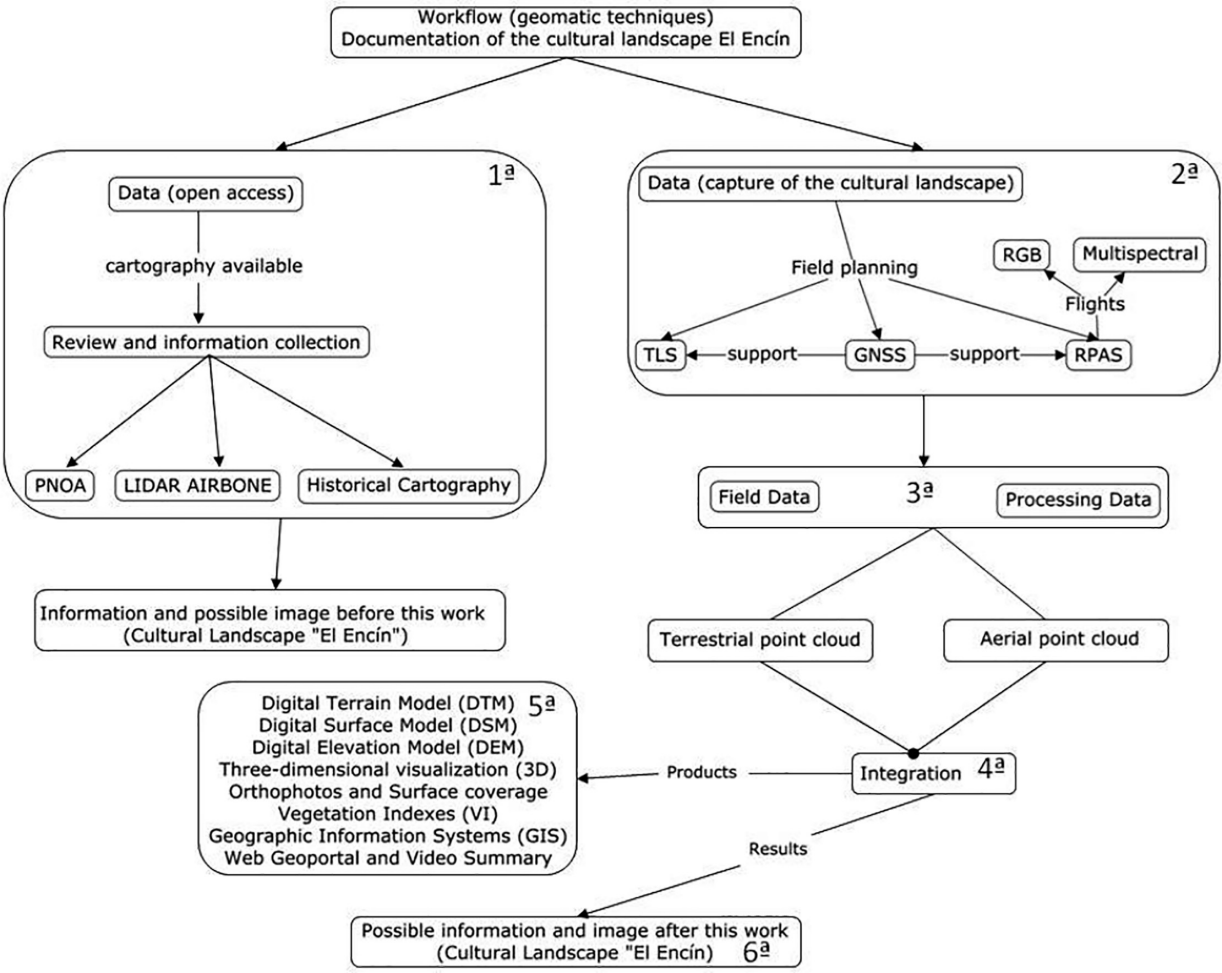
Scheme and phases of the proposal methodology.

The second phase (Phase 2) is the fieldwork to compile the data and capture the information using verified geomatic techniques and instruments: in this case, two UAV flights and a complementary 3D survey obtained by TLS. GNSS techniques were used as control in both cases and as a basis for geo-referencing both point clouds (Fig 3).

**Fig 4:**
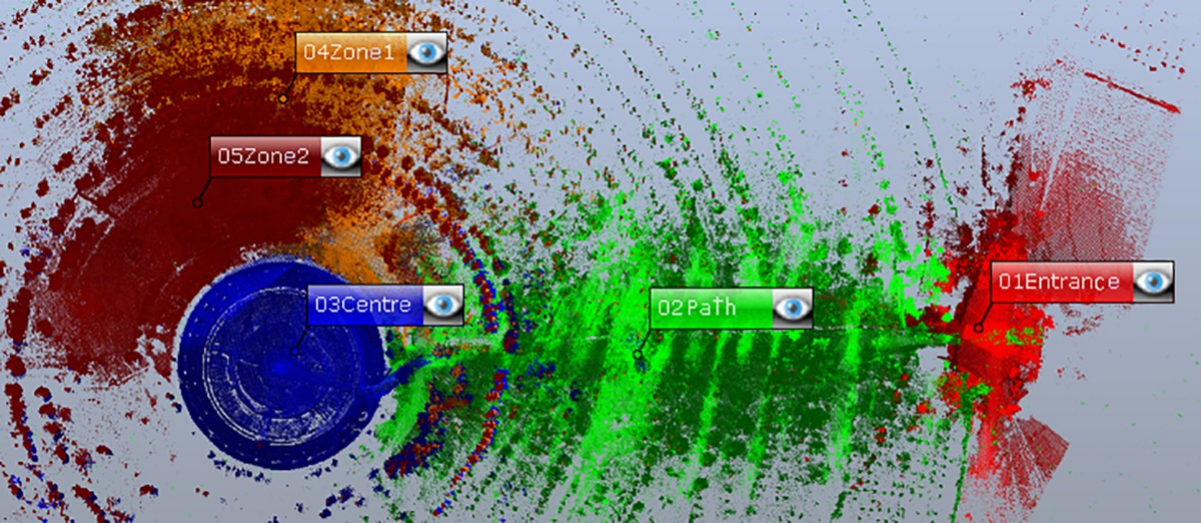
Distribution of zones in the space in the 3D survey. Zone 01, entrance: 9 scans. Zone 02, path: 10 scans, Zone 03, centre: 5 scans, Zone 04: 13 scans and Zone 05: 10 scans. Total 47 scanning stations.

The TLS data were processed by first grouping the scans into five zones based on their homogeneity and the characteristics of the vegetation in the garden (Fig 4). The point clouds are automatically filtered based on the distance and quality of the points. The same scanner took colour photographs in four of the five zones selected, and the last zone was scanned with no colour to reduce the capture time and increase the density of the points to verify the final 3D model. The colour information from these photographs is added to each of the swept points, resulting in a TLS point cloud in the form of a 3D model with a 3D XYZ position, intensity and RGB colour.

Two methods were used to register the scans: the first zone (entrance) was registered cloud to cloud to capture and integrate the height of the building; and the rest of the zones were registered by spheres. Five spheres were plotted to move through the garden: two in front of the scanner position and another two behind, while a third was free to fill in the areas that were more difficult to adjust. Fig 4 shows the path taken inside the garden.

In the third phase (Phase 3) the captured UAV aerial data and TLS data are calculated and processed separately. The UAV data were processed with PhotoScan software to generate the 3D model, DTM (digital terrain model), DSM (digital surface model) and various orthophotos; and with Pix4D software to calculate the various normalized difference vegetation indexes (NDVI or NDRE). The Faro Scene software was used for the TLS data.

In the fourth phase (Phase 4), the data from both platforms are integrated: the UAV images were processed by aerial triangulation with GCPs followed by an automatic point correlation to produce an absolute and accurate 3D point cloud. The TLS scans were first registered in one pointcloud which was subsequently transformed and adjusted using several GCPs. Following this methodology, the two point clouds were merged into the same reference system.

The fifth phase (Phase 5) of this workflow is the map outputs from the research, which are specific to the atomic garden: digital surface model (the result of the arboreal and the ground parts), orthophoto, NDVI vegetation image and the 3D model of the tree cover.

The sixth phase (Phase 6) is the final documentation generated from all the previous phases, and which, once duly organised, offers a precise image and a real and dynamic three-dimensional visualisation of the atomic garden in the study: 360° images and a point cloud for the garden. This information is then integrated in the geoportal and in visualisers so the measurements, surfaces, visual analyses and other elements can be used directly in web environments.

## 3. Results

The end result of the work is the product of the merged information captured for the documentation of the El Encín atomic garden in Madrid (Spain), as described in the second, third, fourth and fifth phases (Fig 3).

### 3.1. Results of the captured data

The GNSS data serve as control for the geo-referencing by the various geospatial technologies, UAV flights (RGB and multispectral) and TLS, as shown in Table 3. The result is a RMSE of 8 mm horizontally and 10 mm vertically (1), which is the accuracy required for geo-referencing the various data sources.

$$\mathrm{RMSE}=\sqrt{\frac{\sum {\left(P_{\mathrm{TLS}}-P_{\mathrm{REF}}\right)}^{2}}{n}}$$ (1)

The photogrammetric adjustment obtained accuracies of 2.60 cm in planimetry and 1.80 cm in altimetry (Table 4). The 3D point cloud was derived from this photogrammetric UAV flight using Agisoft–PhotoScan software.

**Table 4:** Accuracy report for the UAV photogrammetric flight adjustment in mm.

Ground Control Point Adjustment					
Name	XY_error	Z_error	XYZ_error	Proy.	Error (px)
1	0.021	-0.004	0.022	12	0.904
2	0.005	0.001	0.005	8	0.815
3	0.017	-0.006	0.018	17	1.717
4	0.034	0.016	0.037	10	0.664
5	0.057	0.038	0.069	7	0.254
9	0.034	-0.004	0.034	28	1.465
10	0.013	0.007	0.150	12	0.416
C7	0.015	0.040	0.039	33	1.337
T4	0.030	-0.012	0.032	38	1.903
T5	0.033	-0.010	0.034	33	1.402
T6	0.023	-0.006	0.024	37	1.565
**RMS**	**0.026**	**0.018**	**0.031**	** **	**1.436**

Table 5 shows the accuracies for the different groups and the complete model with all the TLS scans. The result of the TLS point cloud was adjusted with the GNSS control points as explained in the following section on integration/merging.

**Table 5:** Accuracy report for the TLS adjustment in mm.

	Max.	Mean	Point <8mm	Point >20mm	Minimum	Obs.
	point error	point error			overlap	
01 Entrance	4.9	3.1	25%	10%	14.9%	Exterior orientation
	Max.	Mean	Max.	Mean	Max.	Mean
	dist. error	dist. error	horiz. error	horiz. error	vert. error	vert. error
02 Path	3	1.2	1.9	0.7	2.3	0.8
03 Centre	4.6	2.2	3.5	1.4	3.3	1.5
04 Zone1	4.3	1.5	1.8	0.7	4.3	1.2
05 Zone2	2.8	1.3	2.3	0.9	2.2	0.8
Total	9.8	1.6	4.6	1	9.2	1.1

### 3.2. Results of the data integration

The two pointclouds are homogeneous in their absolute coordinates and are derived from the work carried out with the GCP, based on the markers for the metric control of the UAV images that were measured and identified with the TLS clouds. The precision of both clouds implies that the 3D merger has a maximum accuracy of 2.6 cm in planimetry and 3.1 cm in altimetry, given by the UAV point clouds.

The resolution of the UAV point cloud was 387 points per m^2^ in the more open areas (with lower vegetation density) and declined from the treetops to the ground area. The 3D model generated by TLS had a resolution of 22,800 points per m^2^ in the ground area and gradually lost resolution towards the treetops or when the laser scanner capture encountered concealed elements in the areas of greatest vegetation density.

The final result was recorded in Laser File Format Exchange Activities–ASPRS (LAS), which meets the requirements for the characterisation of the vegetation described above, namely position X, Y, Z, intensity and colour.

The UAV and TLS point clouds were merged based on vegetation density and tree height, and included UAV points with a height of over 3 m, at a distance of over 20 m from the trajectory of the stations made with TLS, or within the areas marked as exclusive to UAV. A good example of these points is the open area in the centre of the garden (the centre of the atomic radiation of the plants) which is devoid of obstacles and therefore ideal for defining with the UAV cloud. The TLS points were included for zones with a height of less than 3 m or at a distance of less than 20 m from the line of stations made with the TLS. The accuracy of the point clouds is as indicated above, namely 5 cm in planimetry and 3 cm in altimetry, and the resolution is a combination of 387 and 22,800 points per m^2^ according to the filters described.

**Fig 5:**
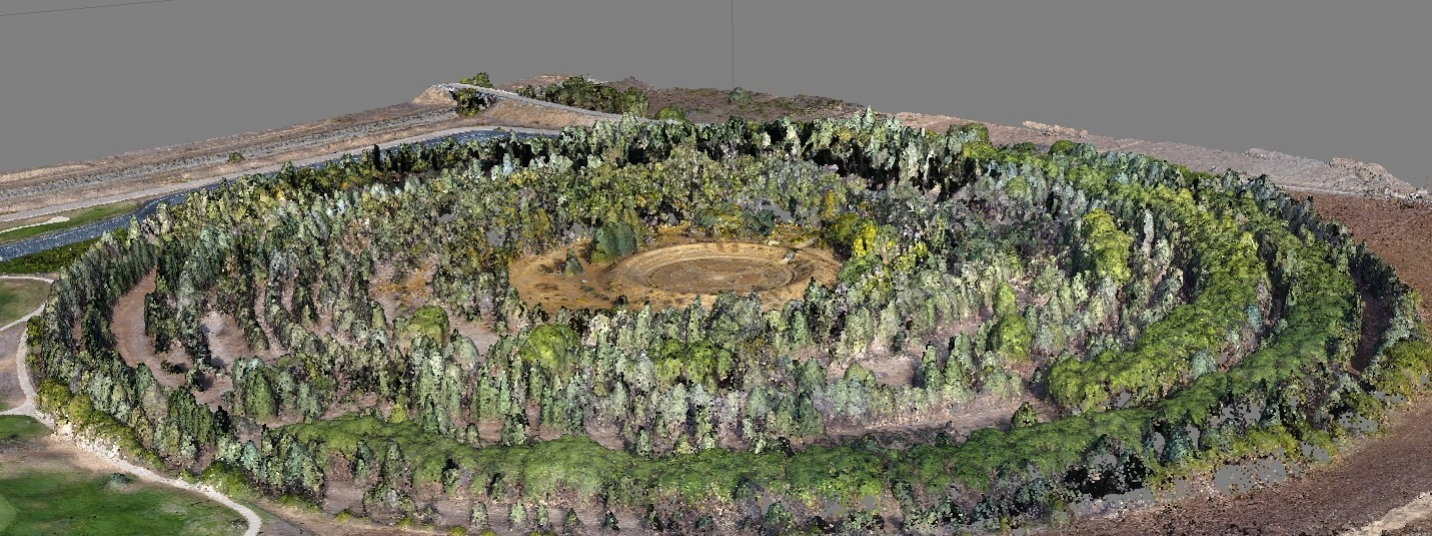
3D mesh model with a resolution of 1.4 faces (triangle texture)/cm^2^.

The following additional outputs were obtained:

From the photogrammetric process:

- Colour orthophotography with GSD of 3.6 cm.
- Digital terrain models (DTM) with a resolution of 6 cm/px.
- 3D mesh models with a resolution of 1.4 faces (triangle texture)/cm^2^ (Fig 5).
- UAV point cloud with a resolution of 387 points/m^2^. This model is only integrated with the TLS when very high resolution or accuracy is required.

From the merger of the photogrammetric process and TLS to obtain the point cloud:

- Integrated 3D point cloud models in LAS format, with 374 points/m^2^ in the zone covered by the UAV platform and 22,800 points/m^2^ in the zones covered by TLS.
- The different data–DTM, DSM, 3D and orthophotos–are stored in a cloud solution.

From the vegetation index obtained from the multispectral camera installed on the UAV platform:

- Orthoimage with NDVI index.

**Fig 6:**
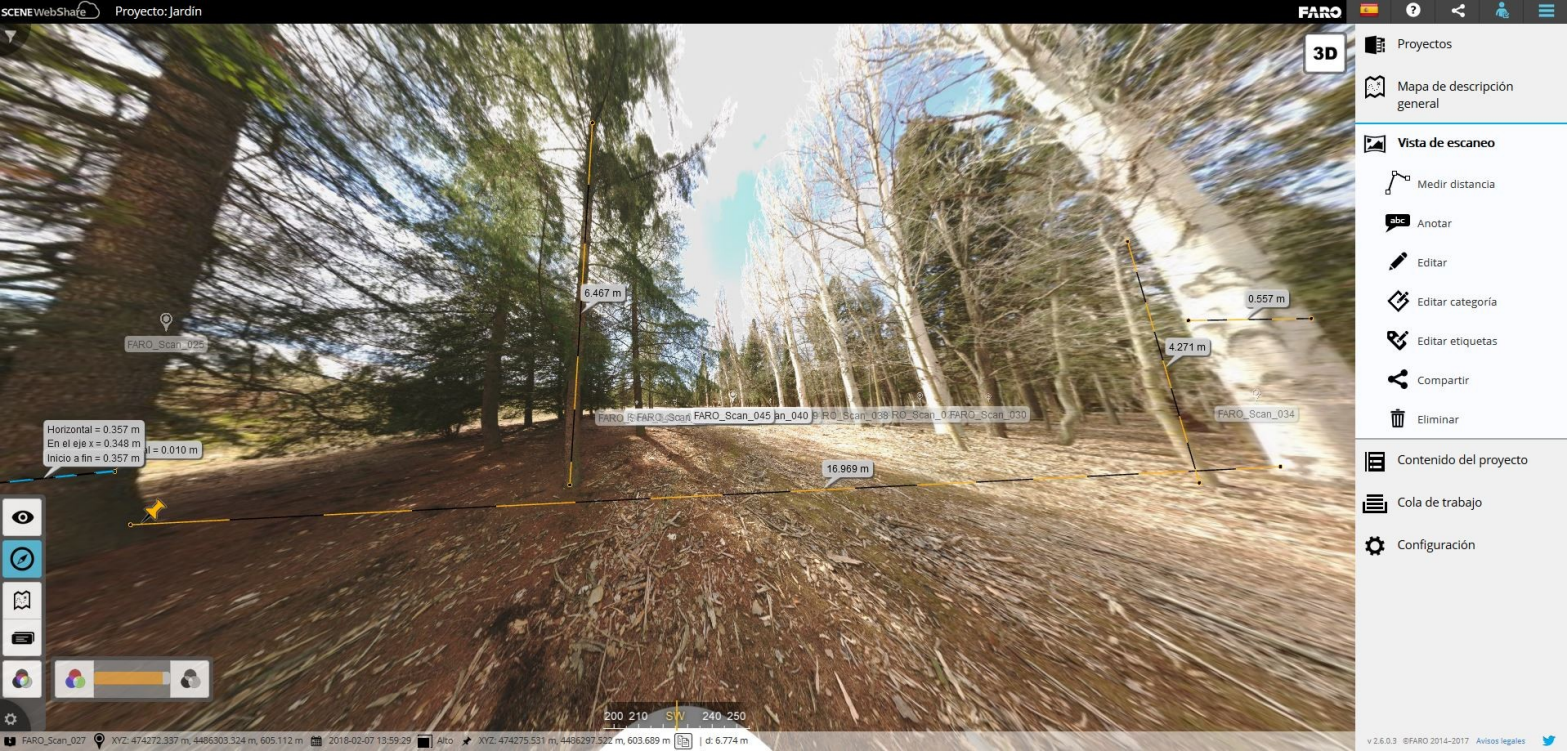
Scene WebShare geoportal. Monitoring the cultural landscape and access to the final image, geovisualisation of the project.

The global output of the research is the integration of the geo-information in geoportals such as Building Information Geospatial Modelling (BIGM), Scene WebShare (Fig 6) and Micasense Atlas. The proposed methodology allows this information to be accessed and shared in the cloud (Internet) by specialists assigned to the project, and openly disseminated to users via geoportal websites.

## 4. Discussion

The background analysed in the first phase of the proposed methodology highlights the lack of metric details and spatial perception of the study zone, producing an inaccurate image. A model for action is proposed which–as explained in Phases 5 and 6 –meets the requirement and offers a highly detailed final image.

The TLS procedure used for massive data capture in this study offers a greater level of detail in the graphic representation of leaves, branches and lesser vegetation compared to other techniques such as terrestrial photogrammetry [53].

**Fig 7:**
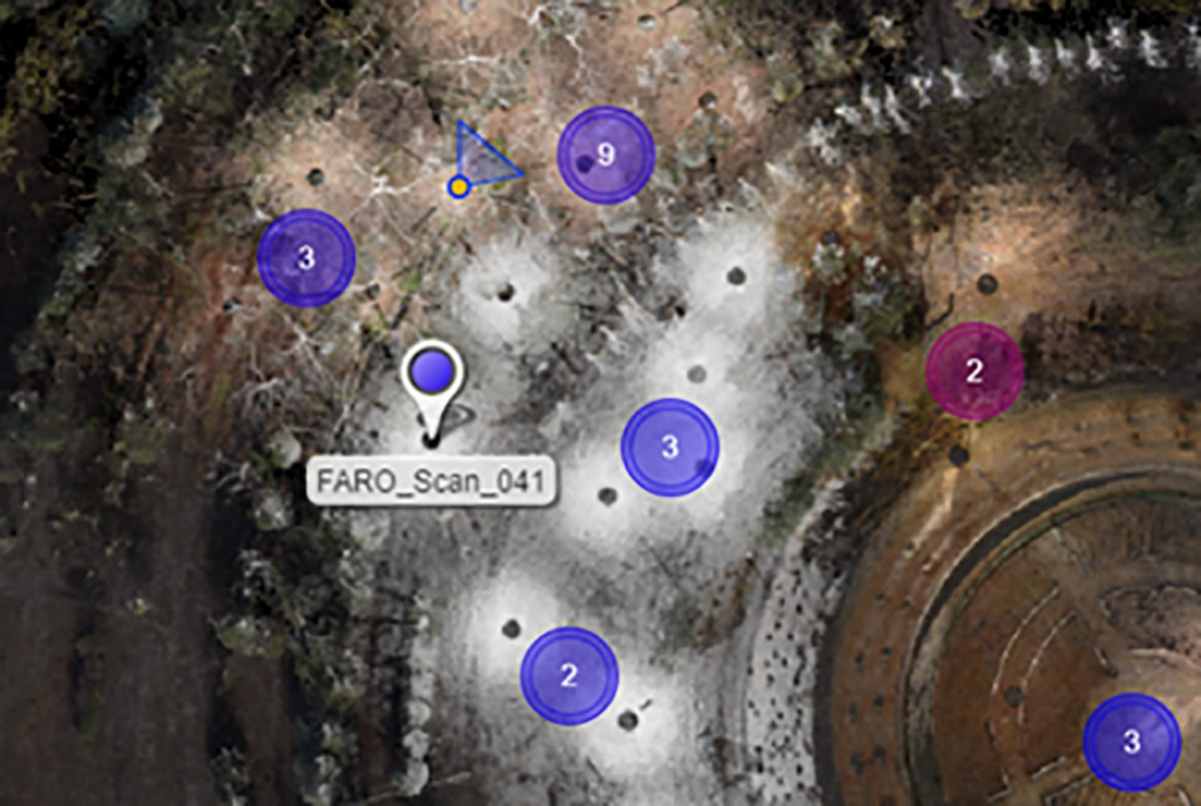
Point-cloud comparison in dense areas. Left: TLS; right: UAV.

One limiting factor in obtaining these digital surface models (DSM) is the difficulty of ensuring the accuracy of the 3D model on clearly defined surfaces such as tree trunks, soil, embankments or buildings, compared to areas with vegetation in a herbaceous or shrubby phase [54]. However, the method is highly suited to assessing this type of vegetation in terms of both its degree of accuracy and the quantification of volumes in the aerial part of the trees (Fig 7).

The map products obtained–DSM, orthophoto and vegetation index (NDVI)–are incorporated into a GIS structure for the optimal cataloguing and inventorying of this type of cultural landscape. The systematic integration of this information on GIS platforms improves the capacity of analysis as it allows managers to modify the point of view from which they study the landscape [55].

Phase 6 consists of the information and image after the study and includes the final documentation generated in all the previous phases. The result is a precise image and a real and dynamic three-dimensional visualisation of the atomic garden in the study.

Finally, thanks to the tools described in Phase 6 of the methodology–geodata/geoportal, web cloud visualisation (click here) and video (see the S1 Video)–users can manage and enhance the final image of the atomic garden in the study as an alternative to traditional methodologies based on photographic reportage and surveys.

## 5. Conclusion and outlook

The results of this work demonstrate that although excellent results can be achieved with high-quality exploration and scanning equipment, a high degree of accuracy can also be obtained with less expensive and complex equipment. Indeed, in terms of accuracy and integrity, the point clouds created from Phantom UAV have proved adequate for documenting, archiving and visualising cultural characteristics.

This lower-technology solution offers significant savings in equipment and training costs, while providing data of sufficient quality to document the cultural landscape in the study.

However, areas with dense vegetation presented complications due to the lack of visibility and penetration of the UAV point cloud near ground level, which hindered the detection of tree trunks.

Another finding is that at heights near ground level, the noise (measuring errors) caused by a lack of visibility due to shadows or concealment in certain images undermines the effectiveness of the UAV model, making the TLS point integration a more suitable option. This is overcome in the UAV-TLS model by delimiting the point clouds of both systems to the highest possible accuracy for each one. The TLS is more effective in closed, low-lying or open areas near the trajectory used to capture the terrestrial points. This methodology confirms the advisability of combining points from both sources depending on the height above ground, tree cover density, light and vegetation height, as proposed in Phase 4.

The definition of the point clouds and the geospatial databases can be considerably improved by combining UAV and TLS technologies, where the limitations of each one are perfectly complemented in the final integrated visualisation. This definition and precision extends to all corners of the garden in the survey, and allows the use of metrics with high internal accuracy when measuring distances at ground level and in aerial zones.

The methodology in this work significantly improves the capture of information on the physical manmade environment by obtaining different information layers: orthophotos, digital surface models, point clouds in LAS format, etc. This geo-referenced data field allows the creation of a two-dimensional model (2D) that represents both the plan and the section of any part of the atomic garden, and a three-dimensional model (3D) to assess spatial conditions. Finally, it includes a time model (4D) for reading the transformation of the atomic garden through subsequent information captures; in other words, by obtaining information in separate time periods it can be seen, using a dynamic three-dimensional model updated for different years, which areas of a cultural landscape require protection, restoration and/or conservation. Hence instead of a graphic representation based on traditional 2D models, we can obtain a 3D and 4D model that effectively highlights the sensitive visual issues that decisively affect the perception of these cultural landscapes. The methodology provides a model offering immersive knowledge supported by virtual reality, augmented reality and 360° video, and integrated information to improve the identification and spatial dimension at various scales and degrees of virtual reality; and, by incorporating time as the fourth dimension, time-sensitive information can be periodically monitored as an effective instrument for the preventive conservation of this landscape. The methodology improves the management and conservation of cultural landscapes by providing 3D models for different time periods, seasons of the year, or yearly intervals. The authors are currently working with this methodology in other sites such as the Jardin de la Isla in Aranjuez, and recommend this approach to other researchers, since our experience demonstrates that the combination of capture techniques improves the preservation of cultural heritage.

## Supporting information

S1 Video Visual summary of the atomic garden and project methodology. (MP4)
